# Lung and diaphragm ultrasound as predictors of success in weaning from mechanical ventilation

**DOI:** 10.1186/s13089-018-0094-3

**Published:** 2018-06-18

**Authors:** Eva Tenza-Lozano, Ana Llamas-Alvarez, Enrique Jaimez-Navarro, Javier Fernández-Sánchez

**Affiliations:** 1 Intensive Care Unit, Department of Intensive Care Medicine, Elche General University Hospital, Camino de la Almazara 11, 03203 Elche, Spain; 2 Department of Clinical Medicine, Miguel Hernandez University, Sant Joan d’Alacant, Spain

**Keywords:** Lung ultrasound, Diaphragm ultrasound, Weaning, Withdrawal of mechanical ventilation

## Abstract

**Background:**

Lung and diaphragm ultrasound methods have recently been introduced to predict the outcome of weaning from mechanical ventilation (MV). The aim of this study is to assess the reliability and accuracy of these techniques for predicting successful weaning in critically ill adults.

**Methods:**

We conducted two studies: a cross-sectional interobserver agreement study between two sonographers and a prospective cohort study to assess the accuracy of lung and diaphragm ultrasound for predicting weaning and extubation outcome. For the interobserver agreement study, we included 50 general critical care patients who were consecutively admitted to the ICU. For the predictive accuracy study, we included consecutively 69 patients on MV who were ready for weaning. We assessed interobserver agreement of ultrasound measurements, using the weighted kappa coefficient for LUSm score (modified lung ultrasound score) and the intraclass correlation coefficient (ICC) and Bland–Altman method for TI (diaphragm thickening index). We assessed the predictive value of LUSm and TI in weaning outcome by plotting the corresponding ROC curves.

**Results:**

We found adequate interobserver agreement for both LUSm (weighted kappa 0.95) and TI (ICC 0.78, difference according to Bland–Altman analysis ± 12.5%). LUSm showed good-moderate discriminative power for successful weaning and extubation (area under the ROC curve (AUC) for successful weaning 0.80, and sensitivity and specificity at optimal cut-off point 0.76 and 0.73, respectively; AUC for successful extubation 0.78, and optimal sensitivity and specificity 0.76 and 0.47, respectively. TI was more sensitive but less specific for predicting successful weaning (AUC 0.71, optimal sensitivity and specificity 0.93 and 0.48) and successful extubation (AUC 0.76, optimal sensitivity and specificity 0.93 and 0.58). The area under the ROC curve for predicting weaning success was 0.83 for both ultrasound measurements together.

**Conclusions:**

Interobserver agreement was excellent for LUSm and moderate-good for TI. A low TI value or high LUSm value indicates high risk of weaning failure.

**Electronic supplementary material:**

The online version of this article (10.1186/s13089-018-0094-3) contains supplementary material, which is available to authorized users.

## Background

Although widespread use of MV in the intensive care unit (ICU) saves hundreds of lives daily, prolonged MV can lead to increased mortality and morbidity [1–3]. On the one hand, therefore, weaning should be considered as early as possible. On the other hand, however, premature withdrawal can result in extubation failure, which is also associated with increased morbidity and mortality [1, 4, 5].

Several ventilatory indices have been developed for identifying the right time to extubate the patient who has required endotracheal intubation and MV, but none met the criteria required to provide suitably accurate success rates [6]. More recently, lung and diaphragm ultrasound methods have been introduced, assessing pulmonary airway patterns and diaphragm function. Bouhemad [7] was the first author to propose the LUS score for calculating lung aeration patterns in patients with ventilator-associated pneumonia. In later articles, this score was used to predict weaning outcome [8–11], with promising results. Several parameters measured through diaphragm ultrasound have been proposed for the same purpose [11–21]. These include diaphragm thickness, diaphragm movement or excursion during the respiratory cycle [22], and diaphragm thickening or thickening fraction (TI). Although some studies have shown diaphragm excursion and thickness to be of low predictive value in the assessment of diaphragm function [18, 19, 23], a recent meta-analysis corroborates this and the best use of TI to weaning outcome [24].

The data suggest that TI and LUS are good non-invasive indicators of weaning outcome. However, the reliability and accuracy of these studies is limited, mainly due to small sample sizes, inadequate spectra of patients and study heterogeneity [24]. The aim of this study is to assess the reliability and accuracy of lung and diaphragm ultrasound for predicting successful weaning in general critical care patients on mechanical ventilation.

## Methods

### Design

We performed two independent studies: a cross-sectional concordance study between two sonographers (interobserver agreement study) and a prospective cohort study to assess the accuracy of lung and diaphragm ultrasound for predicting weaning and extubation outcome (predictive accuracy study).

### Population

For the interobserver agreement study, we included 50 patients (with or without MV), who were consecutively admitted to the ICU of our hospital from December 2016 to February 2017, and who required a thoracic ultrasound examination for clinical reasons.

For the predictive accuracy study, we included consecutively all patients on MV admitted to the ICU from 15 January 2016 to 15 April 2017 who have signed the informed consent (Additional file 1) and met the following inclusion criteria (1) over 18 years of age; (2) more than 24 h on MV; (3) ready for weaning.

We applied the same exclusion criteria for both studies: (1) spinal cord injury higher than T8; (2) arrhythmias and haemodynamic instability; (3) terminal extubation; (4) pregnancy; (5) pneumothorax, pneumomediastinum, thoracostomy, chest tube or chest injuries that prevent ultrasound; (6) pleural lesions or pleurodesis.

### Measurements/procedures

#### Ultrasound technique

Two sonographers trained in lung and diaphragm ultrasound, according to international recommendations [25] (Additional files 1, 2 and 3), performed the ultrasound measurements. They used a 2–4 MHz convex probe in B mode, as described in other studies [7, 8]. The scoring system adopted distinguishes four ventilation patterns as follows: normal aeration (N; presence of lung sliding with A lines and fewer than two isolated B lines), moderate loss of pulmonary ventilation (B1; more than two well-defined B lines), severe loss of pulmonary ventilation (B2; multiple coalescing B lines) and pulmonary consolidation (C; presence of a tissue pattern). Scores of 0–3 were respectively attributed to the four categories (0 point for N, 1 point for B1, 2 points for B2 and 3 points for C), and for each region the worst visible pattern was recorded. Rather than using the original LUS score, in our study, we applied a modified procedure (LUSm), evaluating four lung regions on each side instead of the standard six. Our intention in making this modification was to avoid having to move the critical patient, thus preventing the associated complications and facilitating the examination process for the operators. We assessed four areas: anterior–superior, anterior-inferior, lateral and postero-basal. The postero-basal area is where most of the pathology of the critical patient according to Lichtenstein [26] occurs. The total LUSm score for all areas ranged from 0 to 24 points.

In the diaphragm ultrasound examination, the sonographers measured diaphragm thickness using a 7–10 MHz linear probe in B mode (Micromax^®^ Sonosite) following the technique described in other studies [11–18, 20, 27]. The right hemidiaphragm was visualised in the zone of apposition, on the midaxillary line between the 8th and 10th intercostal spaces, with the patient in a semi-decubitus position (20º–40º). The diaphragm was viewed in M-mode as a hypoechoic structure between two echoic lines (the diaphragmatic pleura and the peritoneal membrane). The sonographers captured almost three images in M-mode during spontaneous patient breathing, measuring diaphragm thickness at the end of expiration and at the end of inspiration. We made the average of three TI measurements using the following formula: (end inspiratory diaphragm thickness − end expiratory diaphragm thickness)/end inspiratory diaphragm thickness.

#### Interobserver agreement study

Both sonographers took TI and LUSm measurements in the same sample of 50 stable patients, with a time difference of less than 5 h between the two operators. This sample was different from the predictive accuracy study.

#### Predictive accuracy study

In the predictive accuracy study, the patients who were ready to start weaning, according to the international consensus conference criteria [28], the respirator was selected with 8 cm H_2_O pressure support (PS) and 5 cm H_2_O positive end-expiratory pressure (PEEP) and ultrasound and ventilatory measurements were made. The ventilatory measurements are made automatically by the respirator (model: GE DATEX- OHMEDA Engström Carestation). Afterwards, SBT was continued with a T-tube or with 8 cm H_2_O PS and 5 PEEP, depending on the decision of the responsible physician, who evaluated which of the patients successfully passed the SBT, and those who did were extubated. The medical team was blinded to the ultrasound results, and the research team played no role in the patient’s weaning. Weaning failure according to the 2007 international consensus conference [28] is defined as either failure of SBT or failure of extubation. Extubation failure is defined as the occurrence of reintubation, non-invasive ventilatory support or death within 48 h following extubation.

### Statistical analysis

Data were expressed as medians and interquartile ranges (IQR) or percentages. To compare continuous variables, we used the unpaired Student’s *t* test, Mann–Whitney *U* test and Wilcoxon test. For categorical variables, we applied the Chi-square or Fisher’s exact test.

To evaluate interobserver agreement for LUSm, we calculated the quadratic-weighted kappa coefficient (which is comparable to ICC) and for the TI variable we used ICC and the Bland–Altman method.

In the predictive accuracy study, we calculated the AUCs and their corresponding sensitivities, specificities and likelihood ratios (LR + and LR−) at the optimal cut-off points, to determine the predictive value of TI and LUSm for weaning and extubation success. We developed a predictive model using binary logistic regression, with the ultrasound measures (LUSm and TI) as independent variables to predict successful weaning.

We used the StatsDirect v3.0.194 package to perform the statistical analysis.

### Ethical aspects

The research ethics committee of Elche General University Hospital approved the study and all enrolled patients gave their informed consent.

## Results

**Fig. 1:**
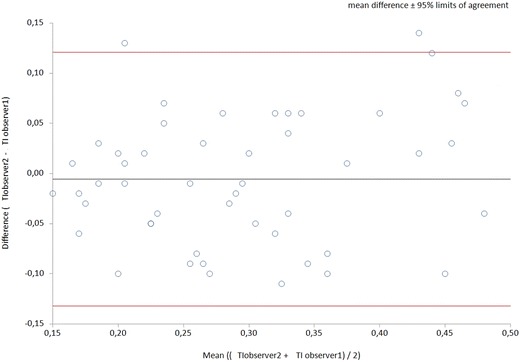
Bland–Altman method for interobserver difference in TI measurement

In the interobserver agreement study, the quadratic-weighted kappa value for LUSm was 0.95 (95% CI 0.92, 0.98), which shows almost perfect interobserver agreement. For the TI variable, we calculated an ICC value of 0.78 (95% CI 0.65, 0.87), showing moderate to good interobserver agreement, and a difference in measurements according to the Bland–Altman method of ± 12.5% (Fig. 1).

**Fig. 2:**
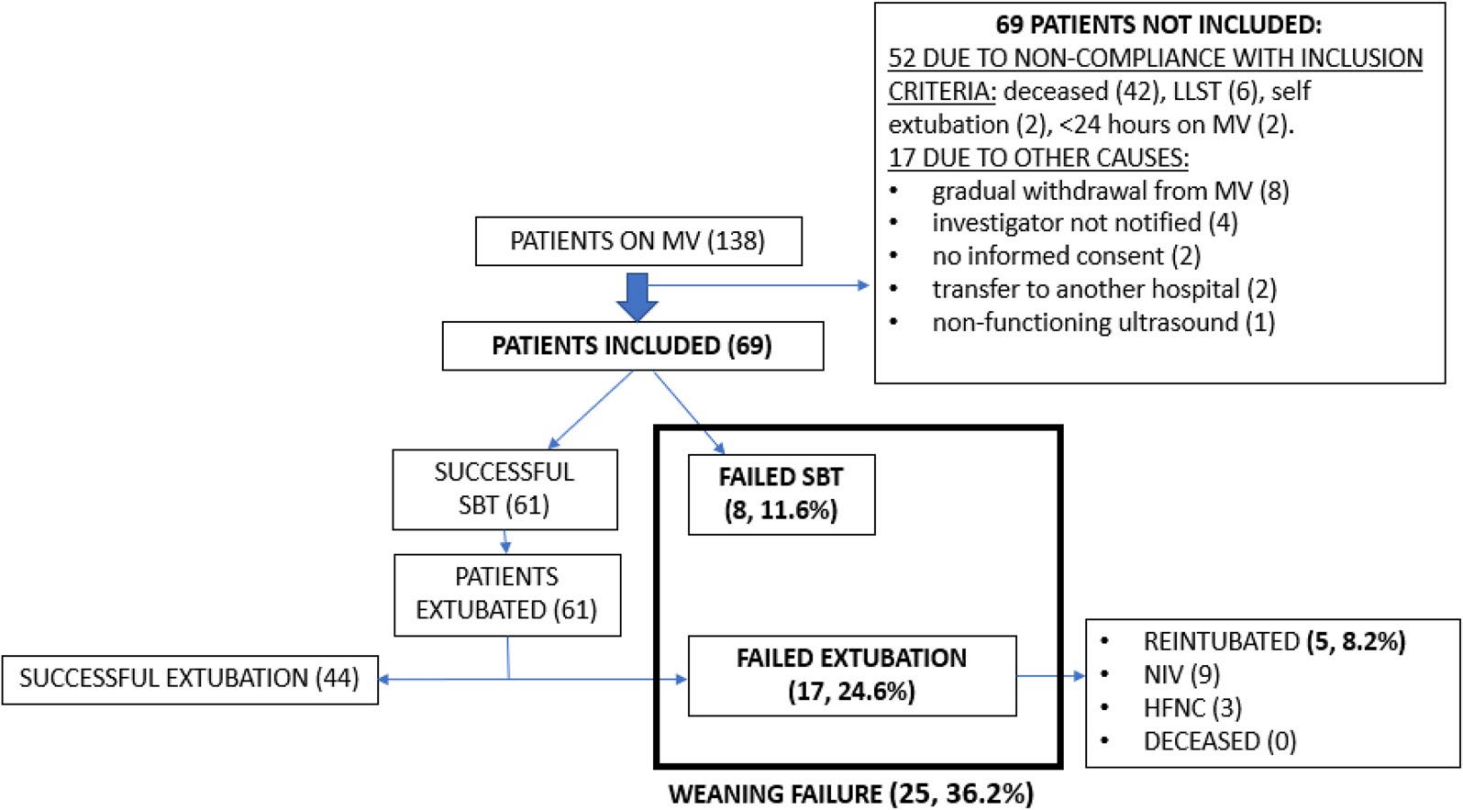
Flowchart of patients

Over the study period, 139 patients underwent MV, of whom 52 did not meet the inclusion criteria (48 deaths before attempted weaning, 2 self-extubations, 2 on MV for less than 24 h) and 17 were not included for reasons beyond the research team’s control (eight withdrawn more gradually from MV, two with no informed consent, two transferred to another hospital, four eligible patients of whom the investigator was not notified, one case of a non-functioning ultrasound scanner). The baseline characteristics of the 69 patients recruited are shown in Table 1. Pressure support was used in 49% of SBTs, a T-tube in 42% and both methods in 9%. Eight patients failed SBT and 61 were extubated, of whom 17 failed extubation. This means that a total of 25 patients failed weaning. Most patients who failed extubation recovered with non-invasive-ventilation (NIV) and high-flow nasal cannula (HFNC); only five patients (8.2%) were reintubated (Fig. 2).

**Table 1:** Characteristics of patients included in the study

Variables	All patients (69)	SW (44)	FW (25)	*P* value
Sex (men)^a^	43 (62.3)	26 (63.4)	15 (62.5)	0.8
Age (years)^b^	66 (53, 78)	65 (53, 78)	69 (64, 78)	0.37
Time on MV (days)^b^	*4 (3, 7)*	*4 (2, 6)*	*5 (3, 9)*	*0.04*
Time SBT-extubation (minutes)^b^	120 (30, 120)	120 (30, 120)	60 (30, 120)	0.15
Mode of STB				
T−T	29 (42%)	17 (38.6%)	12 (48%)	0.14
PS 8 and PEEP 5	34 (49%)	21 (47.7%)	13 (52%)	
T−T + PS 8 PEEP 5	6(9%)	6 (13.6%)	0	
Comorbidity^a^				
Chronic heart disease	18 (26)	14 (31.8)	4 (16)	0.25
Neurological disease	18 (26)	12 (27.3)	6 (24)	0.99
COPD	*13 (18.8)*	*5 (11.4)*	*8 (32)*	*0.05*
Diabetes mellitus	19 (27.5)	12 (27.3)	7 (28)	0.99
Cancer	8 (11.6)	4 (9.1)	4 (16)	0.45
Chronic kidney failure	12 (17.4)	6 (13.6)	6 (24)	0.33
Liver disease	8 (11.6)	3 (6.8)	5 (20)	0.36
Pathology at ICU admission^a^				
Neurological disease	29 (42)	18 (41)	11 (44)	
Respiratory disease	24 (34, 8)	16 (36.4)	8 (32)	
Cardiovascular disease	10 (14.5)	8 (18.2)	2 (8)	0.7
Sepsis	7 (10.1)	5 (11.4)	2 (8)	
Digestive pathology	4 (5.8)	1 (2.3)	3 (12)	
Polytrauma	1 (1.4)	1 (2.3)	0	0.7
LUSm^b^	*6 (4, 8)*	*5 (3, 7)*	*8 (7, 11)*	*0.0001*
TI (%)^b^	*36 (27, 41)*	*38 (31, 45)*	*27 (20, 40)*	*0.003*
APACHE II on SBT day^b^	4 (2, 6)	4 (2, 6)	5 (3, 9)	0.07
VE (L/min)^b^	8.6 (7.5, 10)	8.25 (7.3, 9.8)	9 (8.1, 11.6)	0.13
Compliance (mL/cm H_2_O)^b^	56 (41, 67)	55.5 (43, 69)	59 (40.5, 67)	0.86
PI_Max_ (cm H_2_O)^b^	− 25 (− 23, − 25)	− 25 (− 25, − 18)	− 25 (− 26, − 24)	0.28
P0.1 (cm H_2_O)^b^	1 (1, 3)	1 (1, 3)	1.5 (1, 2.5)	0.47
RSBI (breaths/min/L)^b^	35 (20, 50)	31 (20, 43)	37 (30, 54)	0.16
RR (breaths/min)^b^	17 (15, 20)	17 (14, 19)	19 (16, 22)	0.09
Tidal volume (ml)^b^	400 (450, 585)	508 (452, 572)	500 (440, 600)	0.71
FiO_2_ (%)^b^	30 (28, 35)	30 (28, 35)	30 (28, 35)	0.83
SpO_2_ (%)^b^	98 (97, 100)	99 (97, 100)	97 (96, 99)	0.027
PaCO_2_ (mm Hg)^b^	40 (36, 46)	41 (36, 45.6)	39.6 (37, 45)	0.63
PaO_2_, (mm Hg)^b^	93 (79, 115)	96.5 (83, 117)	92 (74, 115)	0.61
pH^b^	7.42 (7.32, 7.47)	7.4 (7.3, 7.5)	7.4 (7.4, 7.4)	0.93
Lactate (mmol/L)^b^	1.2 (1, 1.7)	1.2 (1, 1.5)	1.3 (1.1, 2)	0.2
ICU mortality^a^	*7 (14)*	*1 (2.3)*	*6 (24)*	*0.005*
Hospital mortality^a^	*11 (16)*	*3 (6.8)*	*8 (32)*	*0.003*
ICU stay (days)^b^	*11 (7, 19)*	*8 (6, 15)*	*18 (11, 21)*	*0.002*
Hospital stay (days)^b^	19 (14, 30)	17 (13.5, 31)	23 (17, 30)	0.08

SW, successful weaning; FW, failed weaning; MV, mechanical ventilation; T−T, disconnection with T-tube with oxygen; PS 8 and PEEP, 5 disconnection with pressure support and 5 cm H_2_O positive end-expiratory pressure; ICU, intensive care unit; LUSm, modified lung ultrasound score; TI, diaphragm thickness index; APACHE II, acute physiology and chronic health evaluation II; SBT, spontaneous breathing trial; VE, minute ventilation; PI_Max_, maximal inspiratory pressure; P0.1, airway occlusion pressure; RR, respiratory rate; RSBI, rapid shallow breathing index; FiO_2_, fraction of inspired oxygen; SpO_2_, oxygen saturation; PaCO_2_, partial pressure of carbon dioxide; PaO_2_, partial pressure of oxygen; ICU, intensive care unit

^a^n (%), ^b^Median (IQR)

If we compare the group that was successfully weaned (SW) with those who failed weaning (FW) (Table 1), we observe that the FW group was associated with more time on MV, more cases of chronic obstructive pulmonary disease (COPD), higher LUSm and mortality, and lower TI and SpO_2_. The median difference in TI and LUSm between the SW and FW groups was 11% and 3 points, respectively.

**Fig. 3:**
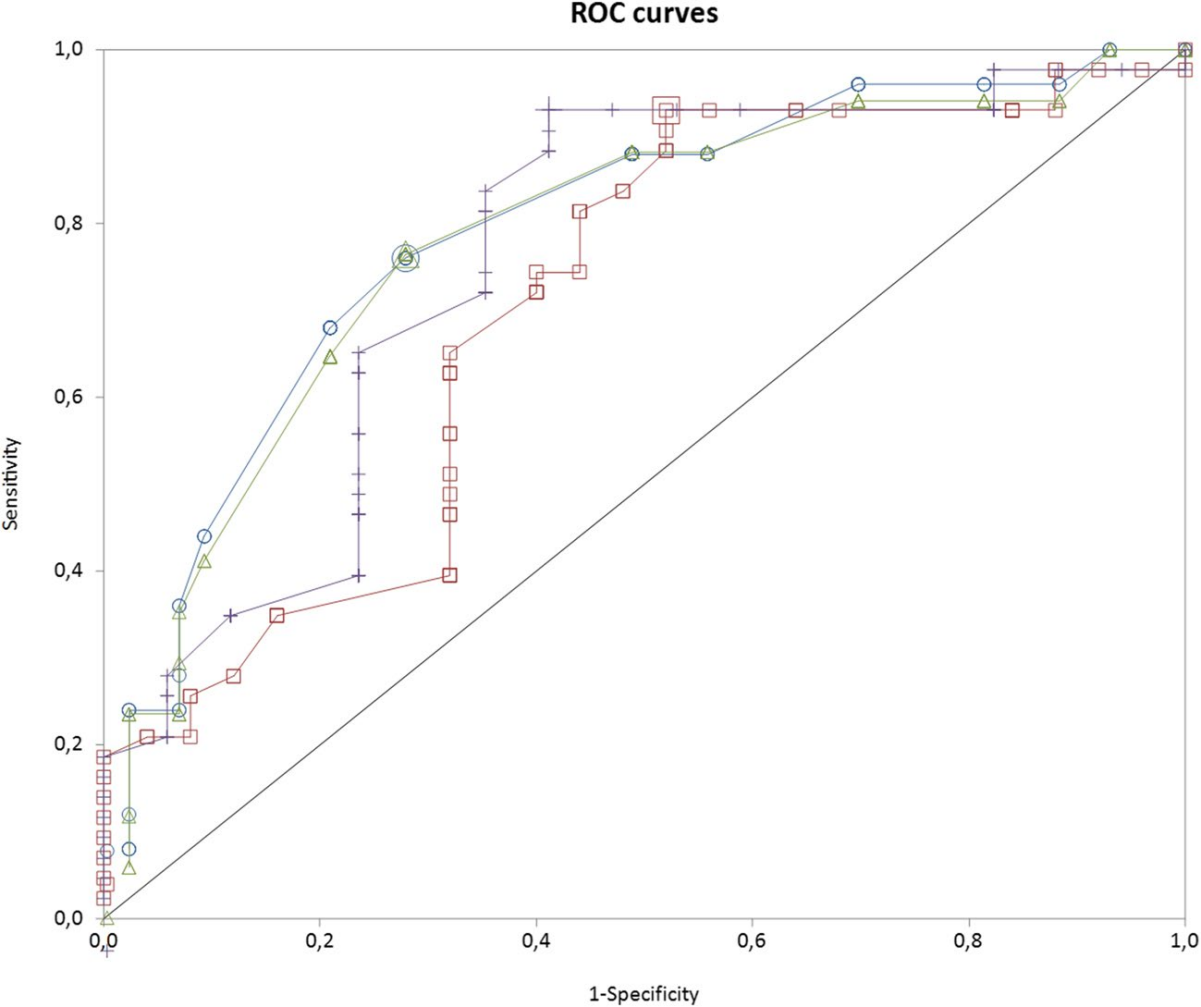
ROC curves for predictive value of TI in successful weaning (SW) (+), in successful extubation (SE) (White square) and LUSm in SW (White circle) and SE (Increment). In SW: LUSm AUC 0.8; TI AUC 0.71. In SE: LUSm AUC 0.78; TI AUC 0.76

**Fig. 4:**
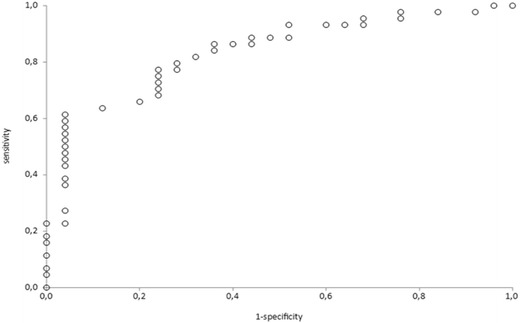
ROC curve for predictive value of TI plus LUSm in successful weaning (SW). AUC 0.83

The area under the ROC curve for predicting weaning success was 0.80 for LUSm (95% CI 0.69, 0.91), 0.71 for TI (95% CI 0.58, 0.84) (Fig. 3) and 0.83 for both (Fig. 4). Table 2 shows the sensitivity, specificity and likelihood ratios at the optimal cut-off points for successful weaning. The area under the ROC curve for predicting extubation success was 0.78 for LUSm (95% CI 0.64, 0.91) and 0.76 for TI (95% CI, 0.61–0.9) (Fig. 3). Table 3 shows the sensitivity, specificity and likelihood ratios at the same cut-off points as those shown in the previous two tables.

**Table 2:** Comparison with other studies of predictive value of TI and LUS for successful weaning

Study (ref)—variable	n	AUC	Sensitivity	Specificity	LR+	LR−	Cut-off point (%)
Binet [9]—LUS	48	0.89	1	0.44	1.80	0	14
Osman [11]—LUS	68	0.94	1	0.94	18	0	12
Shoaeir [10]—LUS	50	0.95	0.83	1		0.17	10
Soummer [8]—LUS	86	0.87	0.68	0.86	4.96	0.37	13
Tenza—LUSm	69	0.80	0.76	0.73	2.8	0.44	7
Ali [16]—TI	54	–	0.96	0.85	6.27	0.04	30^a^
Baess [18]—TI	30	0.65	0.70	0.71	2.43	0.42	30^a^
Blumhof(14)—TI	33	0.86	0.85	0.77	3.67	0.20	20^a^
DiNino [12]—TI	63	0.79	0.88	0.71	3.07	0.17	30^a^
Farghaly [16]—TI	54	0.71	0.9	0.64	2.52	0.16	34.5^a^
Fayed [15]—TI	112	0.93	0.98	0.73	3.66	0.04	29^a^; 24^b^
Ferrari [13]—TI	46	0.95	0.83	0.88	7.03	0.20	36^a^
Jung [17]—TI	33	–	0.61	0.93	9.17	0.42	20^a^
Osman [11]—TI	68	0.89	0.88	1	–	0.12	28^a^
Tenza—TI	69	0.71	0.93	0.48	1.8	0.14	24^a^

AUC, area under the ROC curve; LR+, positive likelihood ratio; LR−, negative likelihood ratio; LUS, lung ultrasound score; LUSm, modified lung ultrasound score; TI, diaphragm thickness index

^a^Right hemidiaphragm; ^b^ Left hemidiaphragm

**Table 3:** Predictive value of LUSm and TI for successful extubation

Variable	AUC	Sensitivity	Specificity	LR+	LR−	Cut-off point (%)
LUSm	0.78	0.76	0.72	2.74	0.33	7
TI	0.76	0.93	0.58	2.26	0.12	24
LUSm + TI	0.83	0.86	0.56	1.97	0.24	

AUC, area under the ROC curve; LR+, positive likelihood ratio; LR−, negative likelihood ratio; LUSm, modified lung ultrasound score; TI, diaphragm thickness index

## Discussion

According to our data, the reproducibility of lung ultrasound is excellent for the variable LUSm and moderate to good for TI. Regarding the prognostic accuracy of ultrasound for weaning outcome, we found that if TI is below 24% or LUSm is greater than 7 points, the patient has a high risk of weaning failure, with an AUC of 0.8 for LUSm and 0.71 for TI. We found similar values for extubation outcome.

Mean time on MV [29, 30], mortality of the patients included in the study (16%) [4], and SBT failure rate (11.6%) [31] was consistent with previously published results. Extubation failure occurred in 24.6% of the patients, a slightly higher proportion than the 10% to 20% reported in a number of other studies [1, 5, 32–35]. Of the 17 patients who failed extubation, only five (8.2%) required reintubation, a lower rate than reported in other studies [36, 37]. This shows that NIV plays a decisive role in reducing the need for reintubation without increasing morbidity or mortality [37, 38]. As such, through conceptually defined as a criteria of weaning failure [28], we consider that recovery with NIV in fact constitutes a success for the patient’s clinical situation. We observed that FW patients were more likely to have COPD. This is a logical finding, as COPD is a risk factor for extubation failure [39, 40]. Of the standard predictors of weaning assessed in our study (PI_Max_, RSBI, P0.1), we found none to be useful. In a study with the largest number of patients performed for the study of weaning predictors [6], about 500 patients, to assess the predictability of many indices as possible weaning predictors (minute volume, respiratory rate, PaO_2_, RSBI, PI_Max_, Maximum expiratory pressure, dynamic respiratory compliance, CROP index), it is observed that none of them has value as a predictor of weaning.

The results obtained for LUSm are consistent with previously reported data [8–11]. Our cut-off point is lower (7 LUSm points) because we assessed eight lung areas, whereas the other studies assessed 12. Our aim was to assess all the areas normally affected in critical patients [26], while simplifying the technique so that the patient did not have to be moved, and the associated complications could be avoided. We therefore consider LUSm to constitute a useful new proposal that is beneficial for both the patient and the operator. For TI, we found a cut-off point of 24% for predicting successful weaning, within the range of values reported in other studies (20–36%) [11–20]. The LUS and TI variables tested in those studies showed a higher predictive value for weaning success (Table 2), but a number of factors may have influenced this result. In those studies, patient selection was in some cases very strict, resulting in a homogeneous sample with specific characteristics (patients with COPD [15], tracheotomised patients with prior weaning failure [13], patients with ICU-acquired weakness [17]). Since these samples already had a higher probability of weaning failure, their results cannot be generalised to the whole population of critical care patients. Other limitations in the reviewed studies included elimination of deceased patients [20], diaphragm ultrasound when the patient was on MV rather than during SBT [20], use of a non-validated probe for diaphragm measurement [18], periods of up to 36 h between the ultrasound scan and extubation [12], and using STB failure rather than extubation failure as an endpoint [13]. Only one of the reviewed studies included a reproducibility study comparing TI measurement by two observers, with results very similar to ours (ICC 0.81) [14].

In our study, we chose not to select specific populations of patients for our study, to obtain a heterogeneous sample and produce generalizable results. The ultrasound measurements were taken within the first minutes of pressure support ventilation, the physicians were blinded to the ultrasound data, no patients were lost to follow-up, and the median time between SBT and extubation was 120 min.

One limitation of our study is the small sample size, which led to imprecise results with broad confidence intervals, especially for TI. Further studies with larger samples of patients are required to establish the true predictive power of these ultrasound techniques.

It should be noted that the interobserver difference in TI measurement (± 12.5%) was greater than the median difference in TI between the SW/FW groups (11%). In our sample, therefore, a degree of uncertainty was associated with this parameter. This is probably because the formula for calculating TI is such that a difference of a tenth of a millimeter in the measurement of inspiratory or expiratory diaphragm thickness has a considerable effect on the result. None of the studies reviewed considers this possibility. We believe the problem can be overcome with better ultrasound equipment and a more effective TI measuring technique, so that the true value of this parameter can be reported.

In the future, in order that results can be compared across studies, we believe a number of items should be standardised: the ultrasound technique, the definitions of weaning failure and extubation failure, the time between ultrasound and extubation, blinding of ultrasound findings, and the protocol. If results are to be applied to the whole population of general critical care patients on MV, a heterogeneous sample should be used. Studies in this domain should also assess reproducibility by measuring interobserver agreement in ultrasound measurements. In addition, it may be useful to consider other parameters, such as time on MV, disease severity, comorbidities, etc., alongside lung and diaphragm ultrasound measurements, to better predict weaning success.

## Conclusions

In our study, interobserver agreement was excellent in LUSm measurements and moderate to good in TI measurements. The TI variable showed a degree of uncertainty for predicting weaning outcome, but overall its predictive value was found to be acceptable. LUSm produced stronger results in this regard. Lung and diaphragm ultrasound are promising techniques for predicting weaning outcome, but more studies are required to verify their reproducibility. These studies should have a standardised design and should assess interobserver agreement of ultrasound techniques. Using a non-specific sample would ensure that results can be generalised to all patients on MV.

## Additional files

**Additional file 1.** Lung ultrasound video.

**Additional file 2.** Diaphragm ultrasound video.

**Additional file 3.** Diaphragm ultrasound video.

## Abbreviations

MV: mechanical ventilation
ICU: intensive care unit
LUS: lung ultrasound score
LUSm: modified lung ultrasound score
TI: diaphragm thickening index
ROC curves: receiver operating characteristic curves
ICC: intraclass correlation coefficient
AUC: area under the ROC curve
SBT: spontaneous breathing
PEEP: positive end-expiratory pressure
IQR: interquartile ranges
LR+: positive likelihood ratio
LR-: negative likelihood ratio
CI: confidence interval
NIV: non-invasive ventilation
HFNC: high-flow nasal cannula
SW: successfully weaned
FW: failed weaning
COPD: chronic obstructive pulmonary disease
VE: minute ventilation
PI_Max_: maximal inspiratory pressure
P0.1: airway occlusion pressure
RR: respiratory rate
RSBI: rapid shallow breathing index
FiO_2_: fraction of inspired oxygen
SpO_2_: oxygen saturation
PaCO_2_: partial pressure of carbon dioxide
PaO_2_: partial pressure of oxygen
APACHE II: acute physiology and chronic health evaluation II
